# Editorial: Mitochondria as a hub in cellular signaling

**DOI:** 10.3389/fcell.2022.981464

**Published:** 2022-08-15

**Authors:** Joshua S. Stoolman, Anna Maria Porcelli, Inmaculada Martínez-Reyes

**Affiliations:** ^1^ Department of Medicine, Northwestern University Feinberg School of Medicine, Chicago, IL, United States; ^2^ Department of Pharmacy and Biotechnology, University of Bologna, Bologna, Italy; ^3^ Max-Delbrück-Center for Molecular Medicine, Berlin, Germany

**Keywords:** mitochondria, signaling, cancer, immunometabolism, neurodegeneration, redox

**FIGURE 1:**
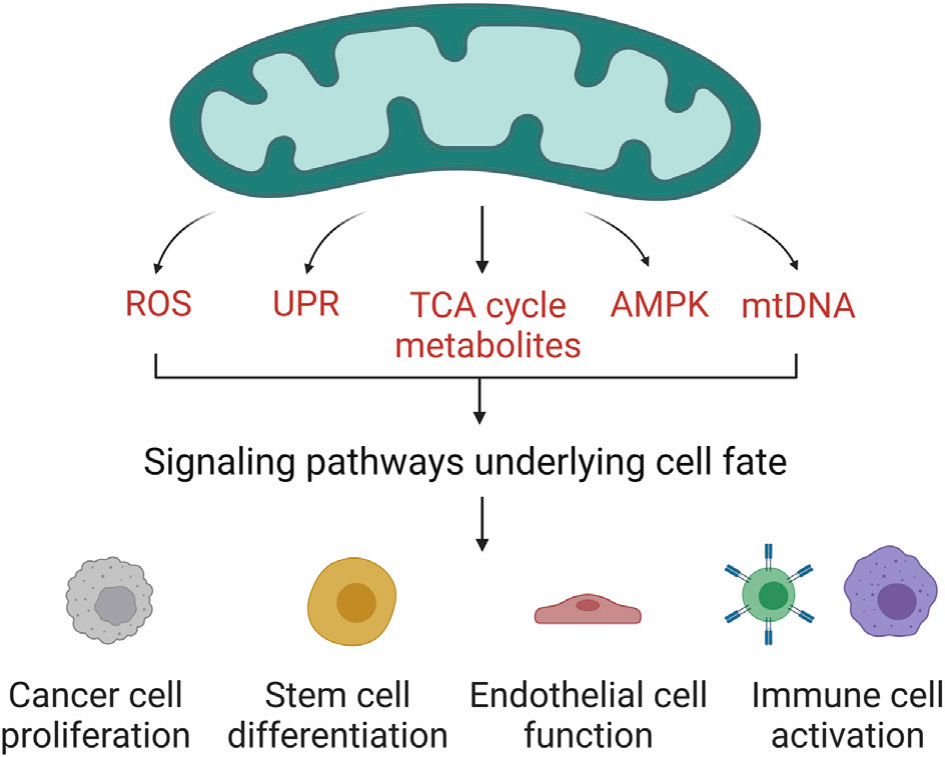
Mitochondrial regulation of cell fate and function. Mitochondria have evolved different channels of communication with the rest of the cell. The release of ROS, mtDNA, or TCA cycle metabolites in certain contexts controls the expression of specific genes. The activation of AMPK or the unfolded protein response upon cellular stress triggers changes in cell fate. Through diverse cell signaling pathways, mitochondria regulate the function of different cell types. Created with https://biorender.com.

Mitochondria are essential organelles for cellular physiology as they are the main source of ATP and metabolites for the synthesis of macromolecules. In the last decade, many studies have added evidence to support a third key role of mitochondria as signaling organelles (Tan and Finkel, 2020). Through signaling, mitochondria regulate a wide range of cellular functions including cancer cell proliferation, stem cell differentiation, and immune cell activation. Evolution generated multiple ways of communication between mitochondria and the rest of the cell (Figure 1). Activation of AMP-activated protein kinase (AMPK) under low energy conditions to activate catabolism or the release of reactive oxygen species (ROS) to activate the hypoxic response are well-known examples (Herzig and Shaw, 2018; Kong and Chandel, 2018). Further, several TCA cycle-derived metabolites are involved in controlling gene expression in part by mediating proteins posttranslational and epigenetic modifications (Ryan et al., 2019; Martínez-Reyes and Chandel, 2020). It is clear that mitochondria play an essential role in regulating cellular homeostasis, however the molecular mechanisms linking mitochondrial function to the expression of specific genes in some contexts remain to be elucidated. We launched this research topic to collect articles that highlight ways in which mitochondrial-dependent signaling determines cell fate and function.

Most tumor types are dependent on mitochondrial metabolism to meet the increased demands imposed by the uncontrolled proliferation that characterize tumor growth (DeBerardinis and Chandel, 2020). Luke Thomas and Margaret Aschcroft in their review piece elegantly covered the importance of the context and tumor type for mitochondrial gene essentiality. The authors discuss the contribution that (nuclear) genome-wide CRISPR-Cas9 deletions screens have made to uncover essential nuclear-encoded mitochondrial genes for cancer cell viability (Tsherniak et al., 2017; Thomas et al., 2021). While the authors highlight the power of this unbiased tool to reveal essential genes, the authors also raise several points of concern about methodological differences in culturing conditions, the sgRNA library used and the cell line(s) selected for the screen, which often hamper the comparison between studies and validation of hits. Overall, the results from the CRISPR screens point towards specific gene essentialities in tumors from different etiologies, which is a critical factor to consider for therapeutic approaches. A tumor type highly dependent on mitochondrial metabolism is hepatocellular carcinoma (HCC), the most common form of primary liver cancers. The use of the antibiotic tigecycline, an inhibitor of mitochondrial protein synthesis, was proven successful in overcoming relapse from tyrosine kinase inhibitors in HCC models (Meßner et al., 2020). The research article by Kabiri et al. included in our collection highlights the potential of targeting augmenter of liver regeneration (ALR), a mitochondrial protein that is part of the mitochondrial disulfide relay system (DRS) to treat HCC. The authors used MitoBloCK-6 to pharmacologically inhibit ALR, which resulted in mitochondrial impairment and decreased cancer cell proliferation. These effects were mostly reversed by addition of the bioavailable hemin b, which demonstrate the importance of mitochondrial iron homeostasis in supporting tumor growth. Moving forward, it will be interesting to see whether MitoBloCK-6 works in HCC *in vivo* models.

Beyond cancer, mitochondrial defects are often the underlying cause of neurodegenerative diseases (Lin and Beal, 2006). In some cases, the downstream effects of mitochondrial dysfunction result in the disruption of cytosolic proteostasis. Jenkins et al., extensively cover this additional channel of communication between mitochondria and the rest of the cell placing the organelle as a determining factor for cellular integrity. The authors propose a model in which under stress conditions, mitochondria mediate the regulation of cytosolic proteostasis by multiple mechanisms including regulation of cytosolic translation, folding capacity, ubiquitination and proteasome degradation and autophagy. These mechanisms operate in combination with the integrated stress response (ISR) and the mitochondrial unfolded protein response (UPRmt). Upon mitochondrial stress, the loss of the mitochondrial membrane potential is a point of no return, which promotes mitophagy and cell death (Ashrafi and Schwarz, 2013). Our topic includes a brief research report by Vasan et al. describing a genome-wide positive selection CRISPR screen that used a combination of mitochondrial inhibitors to uncover genes involved in sustaining a mitochondrial membrane potential (MMP), and therefore ensure cell viability when the electron transport chain is impaired. The screen identified genes involved in protein translation and ATP synthesis as essential for the induction of cell death when cells lose their MMP. Further validation will clarify whether these genes are suitable targets to treat diseases coursing with mitochondrial dysfunction.

ROS are critical signaling molecules linked to the ability of mitochondria to maintain membrane potential. Geldon et al. in their review article, provide a valuable overview of the redox-mediated regulation of mitochondrial homeostasis at multiple levels including biogenesis, dynamics and the assembly of the respiratory chain complexes. The authors suggest that ROS might be sensors of unbalanced ETC activity triggering mechanisms to compensate the deleterious effects derived from it. Uncovering the exact mechanisms mediating this phenomenon might provide additional cues to treat diseases linked to mitochondrial dysfunction.

Our research topic also includes a review article that frame mitochondria as a key regulator of the innate immune system. Bahat et al. highlight the role of mitochondria in translating different metabolic inputs into innate immune responses. During viral infection, changes in the abundance of metabolites, the activity of metabolic enzymes or mitochondrial fusion and fission modulate players of the innate immune response such as the mitochondrial antiviral signaling protein (MAVS) (Ren et al., 2020). Another mitochondrial-related response to viral infections is the release of mitochondrial DNA (mtDNA) (West and Shadel, 2017). The authors provide a comprehensive overview of different mechanisms that lead to mtDNA release to drive effector functions of the innate immune system with an emphasis on their interconnection with changes in the metabolism of nucleotides and lipids. Of note, the activation of innate immune signaling pathways often drives changes in the metabolic status of the cells, which shows the reciprocal relationship between metabolism and the innate immune system. The mechanisms linking metabolic perturbations to innate immune response are not completely understood, but it is clear that elucidating mechanisms of metabolic control on innate immune responses offers new opportunities to improve the clearance of infections. Beyond the effects of mtDNA release in pathological contexts, there is a high interest in the field to understand the physiological effects of mtDNA and explore whether its manipulation can be exploited in the clinic.

Finally, in the original research article by Zhang et al., the proline-rich tyrosine kinase 2 (Pyk2)/mitochondrial calcium uniporter (MCU) pathway is uncovered as a contributing factor for the onset and progression of atherosclerosis. The authors used an apolipoprotein (ApoE) knockout mouse fed with a high-fat diet to model atherosclerosis *in vivo*. Pyk2/MCU expression was significantly increased in the artery wall of atherosclerotic mice. The treatment of these mice with the HMG-CoA reductase inhibitor rosuvastatin, which lowers Pyk2/MCU expression, has a protective effect. Mechanistically, the increased expression of Pyk2/MCU disrupts Ca2+ homeostasis, MMP, ROS production, and apoptotic signaling. These results were recapitulated in an H_2_O_2_-induced endothelial cell damage *in vitro* model. Atherosclerosis (AS) is the leading cause of cardiovascular and cerebrovascular disorders and finding new targets is of high clinical relevance. Future studies will clarify whether specific drugs against Pyk2/MCU pathway are suitable for inhibiting or reversing atherosclerosis.

Overall, our research topic highlights the relevance of mitochondria mediated signaling events in defining cell fate and function in cancer, immune and endothelial cells. Moreover, the articles within our collection add to our knowledge of the effects of disrupting mitochondrial homeostasis in driving different pathologies including neurodegeneration and discusses therapeutic opportunities that place the organelle as a promising target.
